# Risk factors for maxillofacial injuries in a Brazilian emergency hospital sample

**DOI:** 10.1590/S1678-77572010000100006

**Published:** 2010

**Authors:** José Luiz Rodrigues LELES, Ênio José dos SANTOS, Fabrício David JORGE, Erica Tatiane da SILVA, Cláudio Rodrigues LELES

**Affiliations:** 1 DDS, PhD, Emergency Hospital of Goiânia, Health Secretary of Goiás, Brazil; School of Dentistry, Universidade Paulista-UNIP, Goiânia, Goiás, Brazil.; 2 Emergency Hospital of Goiânia, Health Secretary of Goiás, Brazil.; 3 Graduate student, School of Dentistry, Federal University of Goias, Goiania, Goias, Brazil.; 4 DDS, PhD, Adjunct Professor, Department of Prevention and Oral Rehabilitation, School of Dentistry, Federal University of Goiás, Goiânia, Goiás, Brazil.

**Keywords:** Maxillofacial injuries, Epidemiology, Risk factors

## Abstract

**Background:**

Maxillofacial injuries occur in a significant number of trauma patients. Epidemiological assessments are essential to reaffirm patterns, identify new trends and develop clinical and research priorities for effective treatment and prevention of these injuries.

**Objective:**

The aim of this study was to identify the epidemiological profile and risk factors associated with maxillofacial trauma treated at a referral emergency hospital for the Public Health System in the State Capital of Goiás, Brazil.

**Material and Methods:**

A cross-sectional study was designed including 530 patients with maxillofacial trauma, 76% male, with a mean age of 25.5±15.0 years. Data were collected between May 2003 and August 2004 over weekly shift-working periods. Results: The main causes of trauma were traffic accidents (45.7%) and physical assaults (24.3%), and differences in etiological factors were identified according to gender (p<0.001). The distribution of patients according to age and etiology showed significant differences for traffic accidents (p<0.01), physical assaults (p<0.001), falls (p<0.001) and sport injuries (p<0.01). In the multinomial logistic regression analysis (R^2^ = 0.233; p<0.05), age was associated with injury in traffic accidents and falls (p<0.01), sports-related accidents were associated with males (p<0.05), and alcohol consumption with assaults and traffic accidents (p<0.001). Facial soft tissue lesions were found in 98% of patients and facial fractures in 51%.

**Conclusions:**

The significant association of maxillofacial trauma with young males and alcohol consumption reinforces the need for educational strategies and the development of policies for the prevention and reduction of associated damage in this specific risk group.

## INTRODUCTION

Maxillofacial traumas represent one of the greatest challenges to public health services worldwide, because of their high incidence and significant financial cost. They are often associated with morbidity and varying degrees of physical, functional and aesthetic damage^3,7^. Maxillofacial injuries occur in a significant number of trauma patients^12^, and management includes treatment of facial bone fractures, dentoalveolar trauma, and soft tissue injuries, as well as concomitant injuries^2^.

Epidemiological assessments of these injuries are essential to reaffirm patterns, identify new trends, plan and evaluate preventive measures and health policies, and develop priority goals for research. Several studies of the incidence and etiology of maxillofacial traumas have been carried out in countries such as Austria^11,12^, Germany^16^, Iran^24^, Italy^13,31^, Japan^17,18^, Jordan^6,28^, Malaysia^15,29,30^, New Zealand^4,21^, Nigeria^1,25,26^ and the United Arab Emirates^2,19^. Their findings revealed that epidemiological features are related to demographic, socioeconomic, cultural, and environmental factors^2,6^. In general, the main causes are traffic accidents, physical assaults, falls, sports-related injuries and civil wars^12,23^.

Few reports from South American countries are found in the international literature^7^. Epidemiological analysis of maxillofacial trauma is needed and fundamental to assessing health service needs, and for the development of prevention programs and treatment protocols. The aim of this study was to identify the epidemiological profile and risk factors associated with maxillofacial trauma treated at a referral Accident and Emergency Center in Goiania, State Capital of Goias, Brazil.

## SUBJECTS AND METHODS

A cross-sectional study was designed including a consecutive sample of patients with maxillofacial trauma treated at the Oral and Maxillofacial Surgery and Trauma Department of the Emergency Hospital of Goiânia, GO, Brazil, a Public Health System's regional referral care center. The research protocol was approved by the local Institutional Review Board.

Data were collected between May 2003 and August 2004, in at least one weekly shift-working pattern. All incoming patients with maxillofacial trauma were included in the sample of this study. Data were gathered from the clinical and radiographic examinations at the time the patients entered the hospital. All cases were examined by two of the co-authors (FDJ and EJS) and supervised by the chief surgeon (JLRL). In addition, in-patients, patients on recall medical visits or those with non-traumatic maxillofacial injuries, and who declined participating in the study were excluded.

Patients underwent a clinical examination using a specifically designed form developed to investigate the epidemiological features of maxillofacial trauma. Data concerning identification and demographic features of the patient, cause and type of trauma, anatomic site of maxillofacial bone fracture and location of concomitant injuries were collected. Additionally, information about the traumatic event was investigated, such as the time, time lapse from injury to medical examination, alcohol consumption and the use of helmets in the case of motorcycle accidents.

Data analysis was carried out using descriptive statistics, chi-square test and multinomial logistic regression analysis. Significance level was set at p<0.05. SPSS 14.0 for Windows (SPSS Inc, Chicago, IL, USA) was used for statistical analysis.

## RESULTS

The final sample comprised 530 patients with maxillofacial injuries attended in 62 working shifts. Most patients were male (75.8%) and single (70.0%). Age ranged from 0 to 92 years (mean = 25.5; SD=15.0), and peak incidence was in the 21-30 year age, which accounted for 171 cases (32.3%).

**Figure 1:**
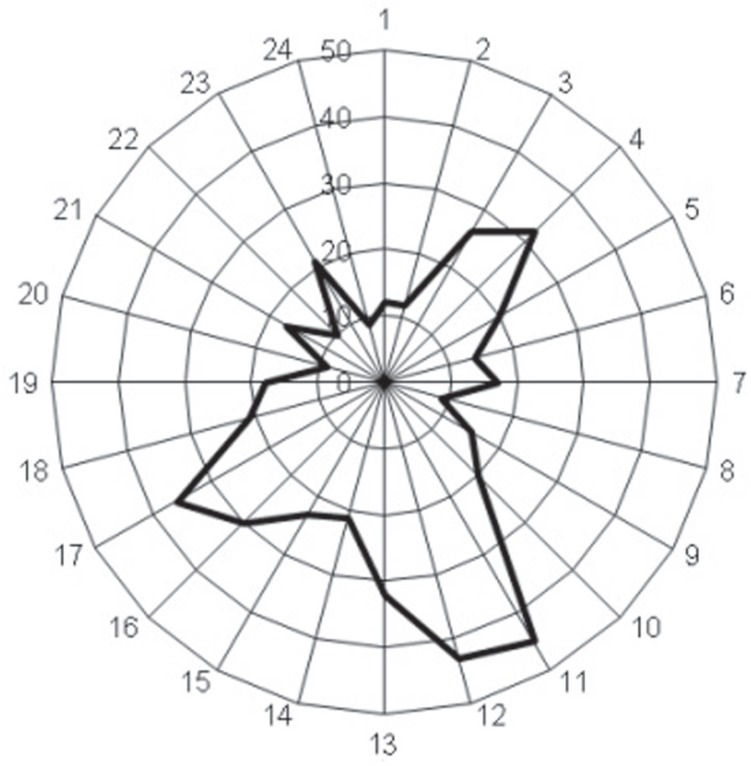
Distribution of maxillofacial traumas according to time when the traumatic event occured

The time lapse from injury to medical examination was up to 12 h in 79% of the cases (n=419). In 21.7% of cases (n=115) the patient was admitted to hospital within one hour after the trauma. In a large number of patients (44.6%), alcohol consumption prior to the accident was reported. Analysis of the time when the accident occurred revealed that the greatest frequencies were at lunchtime, evening and in the early morning (Figure 1).

The most frequent causes of maxillofacial trauma were traffic accidents (45.7%), mainly motorcycle accidents (18.9%) followed by physical assaults (24.3%), falls (17.7%) and sports-related accidents (6.6%) (Table 1). Most motorcycle accident victims were using helmets (76.0%), however 60.5% of them were not fullface helmets.

**Table 1:** Etiological factors of maxillofacial traumas

	**n**	**%**
1 Traffic accidents	242	45.7
1.1 Motorcycle	100	18.9
1.2 Bicycle	73	13.8
1.3 Car	38	7.2
1.4 Vehicle-pedestrian	31	5.8
2 Assaults	129	24.3
2.1 Physical aggression	121	22.8
2.2 Gunshot	8	1.5
3 Falls	94	17.7
3.1 From the standing height or less	76	14.3
3.2 From greater than the standing height	18	3.4
4 Sports-related accidents	35	6.6
5 Other	30	5.7

Distribution of etiological factors according to the type of maxillofacial trauma and anatomic site of facial bone fractures is detailed in Table 2. Concomitant soft tissue facial injuries occurred in 73 patients (13.8%), mainly abrasion (n=43) and edema (n=26). Concomitant bone fracture was diagnosed in 31 patients (5.8%), predominantly in the upper (n=11) and lower extremities (n=10).

**Table 2:** Etiological factors and types of maxillofacial injuries

	**Etiology**					
	**Traffic accidents**	**Assaults**	**Falls**	**Sports**	**Other**	**Total**
1 Facial soft tissue injury (n=514)*						
1.1 Attrition	121	20	19	5	2	167
1.2 Edema	172	110	65	24	22	390
1.3 Laceration	104	45	42	13	11	215
1.4 Epistaxis	19	17	11	6	7	60
1.5 Other	4	3	1	0	0	8
						
2 Facial bone fracture (n=270)*						
2.1 Frontal	8	2	0	2	0	12
2.2 Zygomatic-orbital complex	35	23	7	10	5	80
2.3 Nasal	33	22	9	12	7	83
2.4 Naso-ethmoid orbital	1	0	0	0	0	0
2.5 Maxillary	8	2	0	0	1	11
2.5.1 Le Fort I	1	0	0	0	0	1
2.5.2 Le Fort II	6	1	0	0	0	7
2.5.3 Le Fort III	2	1	0	0	1	3
2.6 Mandibular	36	16	1	2	6	61
2.6.1 Condyle	22	3	0	0	2	27
2.6.2 Angle	7	11	1	0	4	23
2.6.3 Body	9	5	0	1	2	17
2.6.4 Parasymphysis	9	0	0	0	2	11
2.6.5 Symphysis	11	3	0	1	1	16
2.7 Dentoalveolar	47	11	18	2	5	8

* More than one type can be present for each patient.

**Figure 2:**
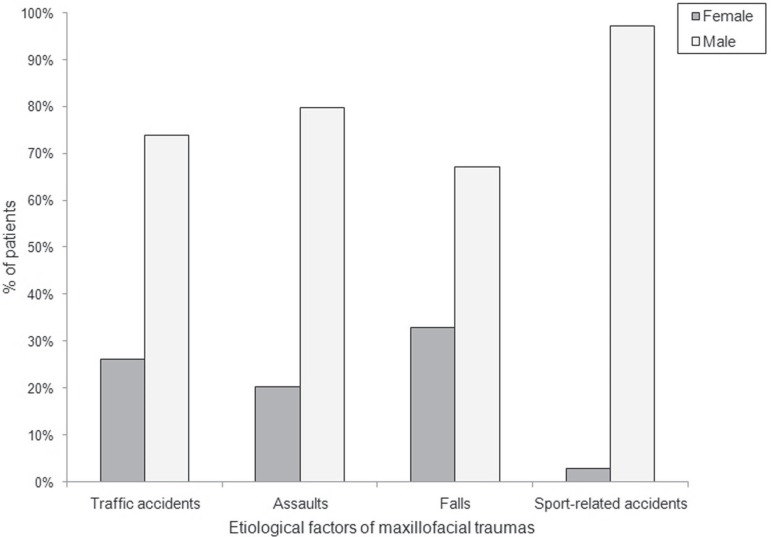
Distribution of patients according to gender and etiology of maxillofacial traumas

**Figure 3:**
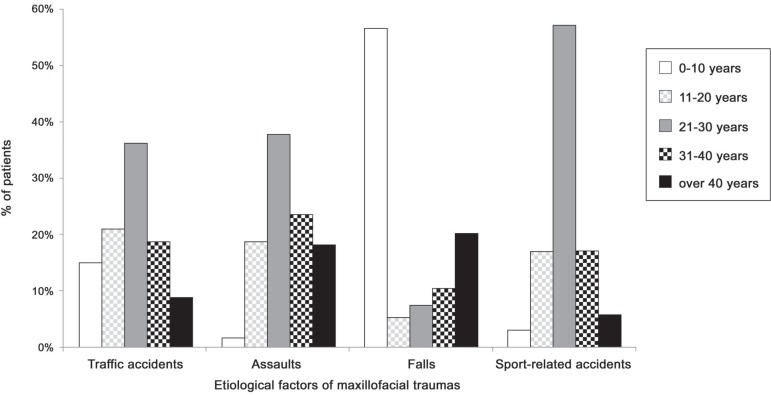
Distribution of patients according to age group and etiology of maxillofacial traumas

Figure 2 shows remarkable gender differences that were identified in the prevalence of etiological factors (Χ^2^=14.26; p<0.001). Cross-tabulation between age groups and etiology of trauma also revealed statistically significant differences for traffic accidents (Χ^2^=17.73; p<0.01), assaults (Χ^2^=31.93; p<0.001), falls (Χ^2^=129.49; p<0.001), and sports-related accidents (Χ^2^=13.70; p<0.01) (Figure 3).

Multinomial logistic regression analysis (Table 3) showed statistically significant associations between age and traffic accidents (p<0.01) and falls (p<0.01), since children and young people were the main victims. Alcohol consumption was associated with assaults and traffic accidents (p<0.001), while sports-related accidents were associated with males (p<0.05).

**Table 3:** Multinomial logistic regression model of the association between etiology of maxillofacial traumas and risk factors.

	**β coefficients (p-value)**			
	**Assaults**	**Sports**	**Traffic accidents**	**Falls**
Age	-0.013 (0.355)	-0.019 (0.266)	-0.039 (0.003*)	-0.042 (0.005*)
Gender	-0.556 (0.278)	+2.362 (0.033*)	-0.644 (0.180)	-0.669 (0.188)
Alcohol consumption	+2.539 (0.000*)	-0.266 (0.705)	+2.014 (0.000*)	+0.643 (0.267)

Age: continuous variable;

Reference categories: Gender - male; Alcohol consumption - yes

R^2^ = 0.233;

* p < 0.05

## DISCUSSION

The higher prevalence of males in maxillofacial trauma (3:1) is well documented in the literature^2,3,5-11,15,19,21,23-26,28,30,33^. Males are at greater risk due to their greater participation in the active population, mainly in non-developed countries, which increases their exposure to risk factors such as driving vehicles, sports that involve physical contact, an active social life and drug use, including alcohol^14,23^. However, over the past 3 decades, an increasing prevalence of trauma has been reported among females, mainly in the under-40 age group, probably due to changes in women's social behavior, including their involvement in non-domestic work, a greater active social life, participation in vehicular traffic and sport as a leisure and health activity^1,23^.

Cultural and socioeconomic features have significant influence in gender prevalence rates of maxillofacial injuries. In countries such as Australia^11^ where women participate widely in social activities, the male-to-female ratios for the occurrence of maxillofacial trauma were reduced by 2:1. On the other hand, Ahmed et al^2^ reported a high prevalence of males (11:1), mostly due to cultural aspects of the United Arab Emirates, where men are usually responsible for work and few women drive vehicles.

The fact that the majority of victims were in the 21-30 age group (32.3%) is also in accordance with other studies^2,5-8,10,15,21,23,24^. This is possibly due to behavioral changes and socioeconomic and emotional conflicts to which these young adults are exposed. This age group is recognized as a phase of great personal independence, social excitement, intense mobility, careless driving on the roads, and exposure to urban violence^8,10,21,24^. The second most affected age group was 31-40 year olds (18.8%). Brasileiro and Passeri^7^ reported that the 21-40 age range represents the economically active segment of society, which is more exposed to maxillofacial trauma risk factors.

Children and individuals the over-40s are less involved in maxillofacial injuries^5,11,18,23,26^. However, the considerable number of patients in the 0-10 age group (17.7%) showed the importance of the development and adoption of specific strategies for the prevention of trauma during the childhood, mainly the prevention of falls, traffic accidents and domestic violence^22,29^.

Traffic accidents were the main cause of maxillofacial injuries, corroborating other international^1,2,6,8,10,15,19,24^ and Brazilian studies^7,23^. Despite governmental regulations about preventive measures such as use of seat belts, helmets and children's car seats, population adherence to preventive measures is variable and, in some cases, minimal^32^. In the present study, 40% of motorcyclists did not wear helmets and most of helmeted victims were not using full-face helmets, with increasing exposure to maxillofacial injuries^30^. Recently, Oginni et al^25^ investigated the risk factors in motorcycle injured Nigerian maxillofacial patients, and reported a rate of helmet use of 3%. These authors stressed the need for the adoption of legislation to make the use of full-face helmets mandatory.

Safer roads, effective law enforcement and public transport policies contributed to a significant decrease in the occurrence of traffic accidents in developed countries over the last three decades^6-10,15,23^. Vehicle accident statistics indicate that the best protection against injury includes safety awareness courses, defensive riding skills and a personal commitment to ride safely at all times^32^.

Physical violence is another increasingly important etiological factor of facial trauma. In countries like Finland, the United States and Sweden assaults has been reported as the main cause of maxillofacial injuries^2,3,5,6,21^. In the present study, assaults were the second most prevalent etiological factor (24.3%), which reinforces the need for the development of preventive programs, aiming to help individuals, organizations, communities, corporations and government agencies plan proactively for the successful mitigation of unexpected violence.

Considering the clinical aspects of maxillofacial trauma, the observed high incidence of nasal and zygomatic-orbital complex fractures is obviously related to the prominent position of these anatomic structures within the facial skeleton, and their greater exposure to external trauma^9,10,21^. The high frequency of concomitant soft tissue facial injuries^12,15,26^ and fractures of upper and lower extremities^1,7^ was also reported elsewhere. Hands and arms are usually used by trauma victims as protective obstacles against a facial injury, while the legs and chest commonly receive direct impact in car crashes or falls^7^.

Depending on the mechanism of trauma, different maxillofacial injury patterns may occur. Nasal and zygomatic-orbital complex fractures are more likely in traffic accidents and physical assaults^7,9,21,23^. Few cases of nasal fractures are reported in maxillofacial trauma studies, since patients are usually referred to otorhinolaryngologists and plastic surgeons^9,21,24^. Traffic accidents were also the main cause of dentoalveolar fractures, especially in bike accidents when security mechanisms are usually neglected^27^. Furthermore, the most common site of mandibular fractures in assault victims was the mandibular angle^17,19^ , and the greater incidence of condyle fractures was observed in traffic accidents^2,19,24^. Frontal and maxillary fractures are usually associated with high-energy traumas, as in traffic accidents^13^.

Alcohol consumption is known to increase crash likelihood^2,3,8,12,21,23,25^, due to reflex reduction and, especially in young people, the abuse of velocity and neglect of safety measures^1^. Alcohol consumption, cell phone use, drowsy or aggressive driving, and driving under the influence of drugs are all important, but preventable, causes of traffic accidents, injuries and deaths^32^. There has been a dramatic, and continuing, drop in alcohol-related traffic crashes, but much more needs to be done to prevent drunk-driving.

Alcohol consumption was also associated with assaults, and considered as the most important factor to trigger acts of violence. Alcohol causes behavioral changes as a result of its psychopharmacological effects, which reduce the ability to make rational decisions and the physical ability to escape or defend oneself^20,21^.

Age was associated with the risk of injuries resulting from falls in two ways: in children^28,31,33^, who are exposed to risk situations owing to their incomplete motor development and greater craniofacial mass to body ratio^28,31^, and in the elderly owing to neuromuscular and motor limitations^12,16^.

Maxillofacial trauma caused by sports-related practices revealed a significant association with gender, which is consistent with several studies that show men to be the group most involved in sports, especially extreme sports or those in which they are more susceptible to accidents, such as football and basketball^4,7,26,31^.

## CONCLUSIONS

The findings of this study indicate the need for the development of emergency protocols, effective educational and communicative strategies and the implementation of policies aimed at preventing and reducing maxillofacial injury and its effects.
